# DeepComplex: A Web Server of Predicting Protein Complex Structures by Deep Learning Inter-chain Contact Prediction and Distance-Based Modelling

**DOI:** 10.3389/fmolb.2021.716973

**Published:** 2021-08-23

**Authors:** Farhan Quadir, Raj S. Roy, Elham Soltanikazemi, Jianlin Cheng

**Affiliations:** Department of Electrical Engineering and Computer Science, University of Missouri, Columbia, MO, United States

**Keywords:** protein quaternary structure prediction, protein complex structure prediction, protein interaction, deep learning, inter-chain contact prediction, distance-based modeling

## Abstract

Proteins interact to form complexes. Predicting the quaternary structure of protein complexes is useful for protein function analysis, protein engineering, and drug design. However, few user-friendly tools leveraging the latest deep learning technology for inter-chain contact prediction and the distance-based modelling to predict protein quaternary structures are available. To address this gap, we develop DeepComplex, a web server for predicting structures of dimeric protein complexes. It uses deep learning to predict inter-chain contacts in a homodimer or heterodimer. The predicted contacts are then used to construct a quaternary structure of the dimer by the distance-based modelling, which can be interactively viewed and analysed. The web server is freely accessible and requires no registration. It can be easily used by providing a job name and an email address along with the tertiary structure for one chain of a homodimer or two chains of a heterodimer. The output webpage provides the multiple sequence alignment, predicted inter-chain residue-residue contact map, and predicted quaternary structure of the dimer. DeepComplex web server is freely available at http://tulip.rnet.missouri.edu/deepcomplex/web_index.html

## Introduction

Proteins interact to form complexes to perform biological functions like gene regulation, signal transduction and enzymatic catalysis (Szklarczyk et al., 2015; Quadir et al., 2021). High-throughput experimental approaches (e.g., yeast two-hybridization) can figure out whether two proteins form a permanent or transient complex; however, these techniques cannot accurately determine the 3D shape of the complex. Biophysical experimental techniques such as X-ray crystallography, nuclear magnetic resonance (NMR), and cryo-electron microscopy (cryo-EM) can determine where and how the proteins interact. These approaches, however, are expensive and time-consuming, and hence can be only applied to a small number of proteins. Therefore, developing accurate computational approaches to reconstruct the quaternary structures of protein complexes has been an important long-standing challenge (Biasini et al., 2014).

During the last 2 decades, many computational techniques, which are fast and inexpensive, have been developed to generate the quaternary structural models of dimers using the tertiary structures of interacting proteins as input (Smith and Sternberg, 2002; Chen et al., 2003; Gray et al., 2003; Comeau et al., 2004; Tovchigrechko and Vakser, 2006; Lyskov and Gray, 2008; de Vries et al., 2010; Hwang et al., 2010). Although the classic *ab initio* docking methods have achieved some success for some protein complexes, according to the last several rounds of Critical Assessments of Predictions of Interactions (CAPRI) (Janin, 2005; Janin, 2002), the general accuracy of these approaches is still low (Lensink et al., 2019). Template-based modelling approaches can predict the quaternary structure of some dimers accurately if good structural templates are available (Biasini et al., 2014; Lensink et al., 2019). However, these methods can only be applied to a small portion of proteins, for which the experimental complex structures of interacting homologues (interlogs) are available (Quadir et al., 2021).

*Ab initio* docking tools perform the energy optimization-based scoring to rank structural models. RosettaDock (Lyskov and Gray, 2008) uses the Monte Carlo approach to dock proteins based on the energy optimization. Some tools like ClusPro (Comeau et al., 2004) and ZDOCK (Pierce et al., 2014) are built upon the Fast Fourier Transformation (FFT) approach to search for the geometric complementarity. Also based on FFT, HDOCK (Yan et al., 2017) can perform both template-based modelling and template free docking between interacting proteins as well as between the interaction of proteins and nucleic acids. A few tools have been able to leverage some inter-chain co-evolutionary information in their protocol. InterEvDock (Yu et al., 2016) performs docking by incorporating co-evolutionary information obtained from the paired multiple sequence alignments (MSAs) of the interlogs, and then selects the top models based on FRODOCK (Garzon et al., 2009) score, SOAP-PP (Dong et al., 2013) score and InterEvScore (Andreani et al., 2013). GREMLIN (Ovchinnikov et al., 2014) and EVcomplex (Hopf et al., 2014; Schelling et al., 2018) can perform the co-evolution based interchain contact prediction using the statistical/mathematical direct coupling analysis (DCA) on the MSA of interlogs. However, due to the limited prediction capability of the statistical/mathematical methods, they can only be applied to a portion of protein complexes with deep MSAs.

Although deep learning methods like convolution neural networks, graph neural networks, residual networks, and transformers, has been used in the computational modelling of protein structures, most focus was put on the development of deep learning methods for intra-chain contact prediction, intra-chain residue-residue distance prediction, quality assessment, and tertiary structure prediction (Zeng et al., 2018; Alquraishi and Valencia, 2019; Baek et al., 2021; Jumper et al., 2021; Quadir et al., 2021; Yan and Huang, 2021). The use of very deep and complex deep learning networks coupled with multiple sequence alignments (e.g., Google DeepMind’s AlphaFold), has significantly advanced tertiary structure prediction. Additionally, recent advances in techniques such as cryo-EM in the form of better electron guns, energy filters, cameras, etc. has led to improvements in the atomic resolution of tertiary and quaternary structures of proteins available in the protein data bank (PDB), and hence, has increased the quantity and quality of data available for training, testing and validation of computational prediction of structures of proteins (Nakane et al., 2020), particularly protein complexes. But, the use of deep learning for prediction of quaternary structures of protein complexes has not been well explored, especially when it comes to heteromeric protein complexes.

Recently, ComplexContact (Zeng et al., 2018) web server was developed for inter-chain contact prediction of heterodimers. It used a deep learning network that was pretrained for intra-chain contact prediction to predict inter-chain contacts of heterodimers from the features obtained using a homology-based, genome-based and phylogeny-based multiple sequence alignment. Another deep learning method, DeepHomo (Yan and Huang, 2021), was developed for inter-chain contact prediction of C2 symmetry homodimers. Also, DNCON2_Inter (Quadir et al., 2021) predicted inter-chain contacts of homodimers by removing intra-chain contacts, with some degree of flexibility, from the contacts predicted from the multiple sequence alignment of a monomer. Inspired from the successful performance of AlphaFold2 (Jumper et al., 2021) in CASP14, recently RoseTTAFold (Baek et al., 2021) was developed which performs end-to-end direct prediction of tertiary structure (atomic coordinates) of proteins directly from multiple sequence alignments using three-track attention-based neural networks. Based on the test on a few complexes, the work also shows the deep learning’s potential of predicted the quaternary structures of dimers and trimers provided the paired multiple sequence alignment of sufficient depth is available. However, despite the recent exploration, few general tools are available to use the inter-chain contact prediction directly to generate the final quaternary structure of the complexes. Therefore, it is necessary to develop a user-friendly, robust pipeline leveraging the cutting-edge deep learning technology to predict inter-chain contacts and use them with the distance-based modelling to generate high-quality quaternary structures of protein complexes.

Here we introduce DeepComplex, an automated web server for ab initio prediction of protein complex structures. DeepComplex employs deep learning techniques to predict inter-chain residue-residue contacts from protein sequences first (Quadir et al., 2021; Zeng et al., 2018; Quadir et al., 2020). It then utilizes a gradient descent-based optimization method to use the predicted contacts together with physicochemical and geometrical information as restraints to model the quaternary structures of interacting proteins rather accurately (Soltanikazemi et al., 2021). DeepComplex provides an easy and convenient way for users to quickly obtain predicted quaternary structures of both water-soluble and membrane-associated protein dimers of any organisms.

## Design, Use and Performance of DeepComplex Web Server

### Server Input

**FIGURE 1:**
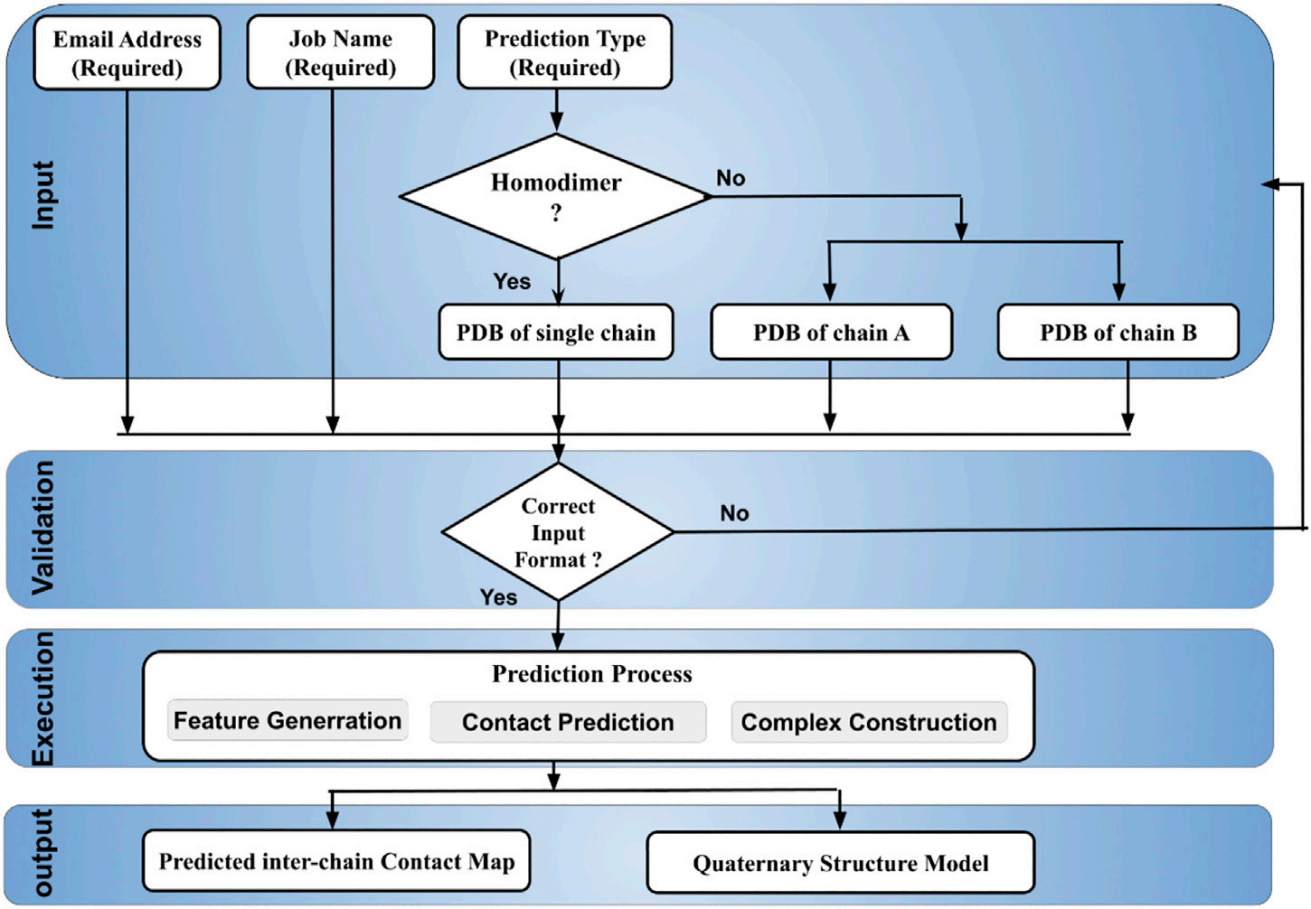
The workflow of DeepComplex illustrating the input, validation, prediction and output.

The DeepComplex web server prompts a user to provide a handful of required inputs to start the prediction process illustrated in Figure 1. Basic inputs like email address and job name are used to identify prediction tasks and send results back to users. Two radio buttons are used for users to choose a prediction type: homodimer or heterodimer. If it is a homodimer, the tertiary structure information of only a single chain in the PDB format needs to be copied into the text box or uploaded as a file. Otherwise, the tertiary structure information of both chains in a heterodimer needs to be provided. Once the job is finished, an email is sent to the email address containing a link to the webpage of the prediction results. The results can be viewed and downloaded at the webpage.

### Server Processing

When a user submits a job, the provided information is first validated and the job is then queued for execution. Once the job is scheduled to run, the protein sequence is extracted from the input structure file and is used to generate the multiple sequence alignment from which the residue-residue co-evolution features as well as other features such as secondary structure and solvent accessibility are generated. They are used for the deep learning-based inter-chain contact prediction (Quadir et al., 2020). These inter-chain contacts are then used to generate the distance restraints for the gradient descent optimization method (Soltanikazemi et al., 2021) to predict quaternary structures of dimers.

### Server Output

**FIGURE 2:**
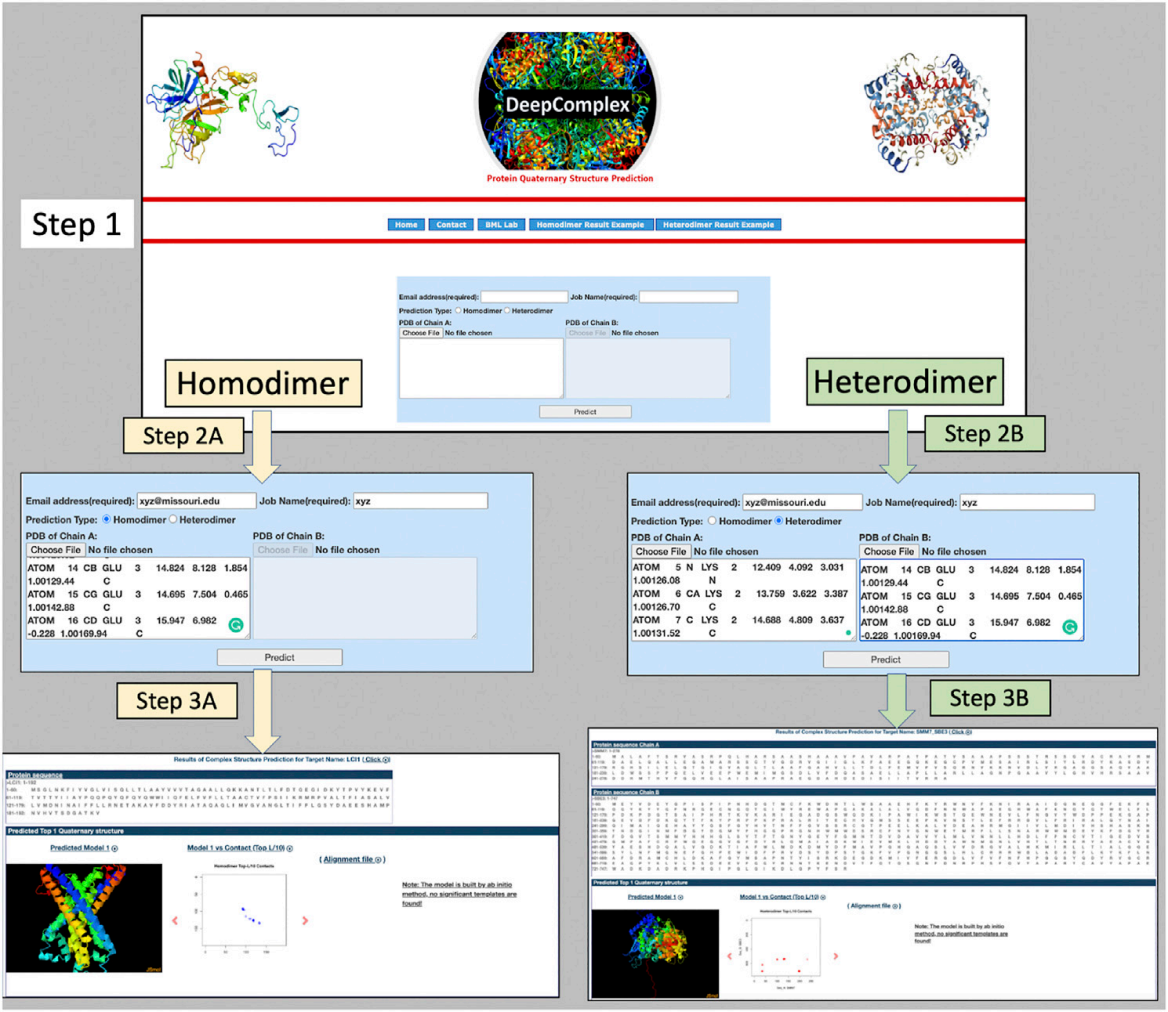
The input and output interface of DeepComplex. Step 1 is the landing page; Steps 2A and 3A are input/output pages for homodimers; and Steps 2B and 3B are input/output pages for heterodimers. In Step 2A, homodimer is selected and the tertiary structure information for one chain is provided; and the output webpage is displayed on Step 3A. Similarly for heterodimer in Step 2B, heterodimer is selected and tertiary structures of the two interacting proteins are provided; and the output is displayed on Step 3B.

On the successful completion of the job, the user is notified via an email containing a link to the output webpage. DeepComplex outputs a comprehensive set of results such as the sequence of the individual chains, the multiple sequence alignment, the predicted inter-chain contact map, and the reconstructed quaternary structure of the dimer. The predicted structure is shown in JSmol (Hanson et al., 2013), which provides a web-based interactive visualization of the complex structure. The visualization of the multiple sequence alignment (MSA) in a separate window is also possible through a click on the “Alignment File” link. All the information in the output page is downloadable individually or as a zipped file (deepcomplex_results.tar.gz). Figure 2 shows the continuous screen shots from the input to the final output for both homodimer and heterodimer cases.

### Server Implementation

DeepComplex is hosted by the tulip.rnet.missouri.edu server. The operating system of the server is CentOS Linux September 7, 2009, which runs on 64-bit 3 GHz AMD Opteron CPU with 16 cores and 64 GB RAM. The front end of the web server is implemented with HTTP, HTML, cgi-bin, and JavaScript. The backend of the server is implemented in Linux shell script, C, C++, JavaScript, Perl, R, PHP and Python. DeepComplex is freely available and does not require any registration.

### Server Performance

The performance of the prediction pipeline of DeepComplex was mainly tested on 115 homodimers from the Homo_Std dataset (Quadir et al., 2021; Soltanikazemi et al., 2021) with predicted inter-chain contacts. The average time taken for a full prediction to be completed for a protein of length of around 500 residues is approximately 477 min with the bulk of the time being for input feature generation. The average TM-score (Zhang and Skolnick, 2005) of the homodimer complex structures generated by the method is 0.76 (average interface RMSD (Lensink et al., 2016) is 7.04 Å; average ligand RMSD (Lensink et al., 2016) is 17.85 Å; and, average percentage of native contacts in predicted model or f_nat (Lensink et al., 2016) score is 30.54%), and for >40% of these homodimers, high-quality structural models with TM-score ≥ 0.9 are obtained (Soltanikazemi et al., 2021). The final quality of the complex structures heavily depends on the precision of the predicted contacts and if precision of the contact prediction is over 20%, in most cases good quality quaternary structures can be built by the system. The distance-based complex structure modelling method was also tested on a dataset of 73 heterodimers with true inter-chain contacts, which achieved an average TM-score of 0.92, average interface RMSD of 0.72 Å, average ligand RMSD is 3.75 Å, and average f_nat score of 90.31% for the reconstructed complex structures. The performance on the heterodimers with predicted inter-chain contacts will be evaluated in the near future. The source code of the deep learning inter-chain contact prediction and the gradient descent optimization used by this web server is available at https://github.com/jianlin-cheng/DeepComplex *.*

## Conclusion

The DeepComplex web server is a convenient, effective, and user-friendly tool for predicting the structure of homo and heterodimeric complexes. It applies deep learning to predict inter-chain residue-residue contacts of a dimer and then uses them to derive distance restraints for a gradient descent-based optimization method to reconstruct the final quaternary structure. DeepComplex is one of the first web servers that can predict both inter-chain contacts via the deep learning as well as quaternary structures of dimeric complexes via the distance-based modelling. It provides a unique tool for *ab initio* protein quaternary structure prediction which is very different from traditional docking methods.

## Data Availability

The original contributions presented in the study are publicly available. This data can be found here: https://github.com/jianlin-cheng/DeepComplex.
